# Auto-thresholding for unbiased electron counting

**DOI:** 10.1093/jmicro/dfaf025

**Published:** 2025-05-19

**Authors:** Julie Marie Bekkevold, Jonathan J P Peters, Ryo Ishikawa, Naoya Shibata, Lewys Jones

**Affiliations:** School of Physics, Trinity College Dublin, College Green, Dublin D02 PN40, Ireland; Advanced Microscopy Laboratory, Centre for Research on Adaptive Nanostructures and Nanodevices (CRANN), Trinity College Dublin, Dublin D02 DA31, Ireland; School of Physics, Trinity College Dublin, College Green, Dublin D02 PN40, Ireland; Advanced Microscopy Laboratory, Centre for Research on Adaptive Nanostructures and Nanodevices (CRANN), Trinity College Dublin, Dublin D02 DA31, Ireland; Institute of Engineering Innovation, University of Tokyo, Bunkyo, Tokyo 113-8656, Japan; Institute of Engineering Innovation, University of Tokyo, Bunkyo, Tokyo 113-8656, Japan; School of Physics, Trinity College Dublin, College Green, Dublin D02 PN40, Ireland; Advanced Microscopy Laboratory, Centre for Research on Adaptive Nanostructures and Nanodevices (CRANN), Trinity College Dublin, Dublin D02 DA31, Ireland

**Keywords:** electron counting, scanning transmission, electron microscopy, single electron events, threshold optimization

## Abstract

As interest in fast real-space frame-rate scanning transmission electron microscopy for both structural and functional characterization of materials increases, so does the need for precise and fast electron detection techniques. Electron counting, with monolithic, segmented, or 4D detectors, has been explored for many years. Recent studies have shown that a retrofittable signal digitizer for a monolithic or segmented detector can be a sustainable and accessible way to enhance the performance of existing detectors, especially for imaging at fast scan speeds. Since such signal digitization uses a threshold on the gradient of the detector signal to identify electron events, appropriate threshold choice is key. Previously, this threshold has been set manually by the operator and is therefore inherently susceptible to human bias. In this work, we introduce an auto-thresholding approach for electron counting to determine the optimal threshold by maximizing the difference in identified counts from a stream with real electron events and a stream with only noise. This leads to easier operation, increased throughput and eliminates human bias in signal digitization. When pixel dwell time becomes shorter than scintillator response time, digitization of the detector signal is needed to avoid artefacts in STEM images. Optimizing the threshold for this digitization process automatically is crucial to achieve high-quality quantitative digitized images, free of human bias for what threshold yields the best digitization.

## Teaser

When pixel dwell time becomes shorter than scintillator response time, digitisation of the detector signal is needed to avoid artefacts in STEM images. Optimising the threshold for this digitisation process automatically is crucial to achieve high-quality quantitative digitised images, free of human bias for what threshold yields the best digitisation.

## Introduction

Scanning transmission electron microscopy (STEM) is a powerful tool for high-resolution characterization of materials [1–3]. In recent years, the scan generators used for STEM have become faster, allowing STEM images to be acquired with increasingly short pixel dwell times down to the tens of nanoseconds range [4–6]. This has enabled the imaging of fast dynamics in materials by acquiring multiple sequential fast frames [4, 6]. However, since most scintillator-based detectors have a decay time in the range 0.1–0.9 µs [7], when selecting dwell times below a microsecond, the detector response time starts to play a critical role in the integrity of the acquired images [8]. In this case, streaking artefacts appear in the image and a damping in Fourier space is seen [7]. In recent years, real-time digitization of the detector signal from scintillator-based detectors has been shown to maintain the integrity of the images acquired since the digitization picks out each electron event only at the rise time of the scintillation [9–10]. As a result, all streaking artefacts are removed and electrons are collected in the correct image pixel only [10].

**Fig. 1.**
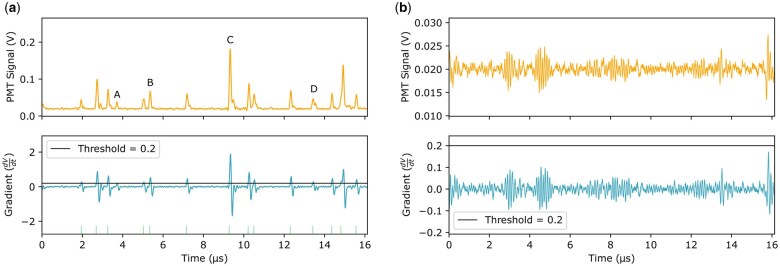
Example 16 µs data streams, extracted from full 80 µs data streams for visibility, from the ADF detector used in this work. (a) Signal stream showing both the raw signal voltage (orange, top row) and its gradient (blue, bottom row) with the threshold (horizontal black line), as well as the resulting digitized signals (green ticks at the bottom of gradient plot). (b) Noise stream where none of the noise is counted as events with the same set threshold Γ = 0.2. The four events highlighted in (a) are: A, an electron event that is detected so weakly that it is not counted in the digitization. B, an event giving a low signal, but still enough to be digitized. C, an electron event giving a very strong signal. Finally, D is an example of coincidence loss where two electrons arrive too close to be separated into two events.

Multi-frame acquisition can also help in the characterization more beam-sensitive materials, since spreading the beam exposure across multiple shorter exposures seems to delay the onset of beam damage [11]. Additionally, advances in phase contrast and retrieval techniques in recent decades have enhanced the detection efficiency, allowing for lower electron doses and expanding the reach of what materials may be characterized with STEM [2, 5, 12, 13]. However, when the electron dose is reduced, accurate detection of every single electron becomes increasingly important to maintain the signal level and the contrast-to-noise ratio (CNR) [14]. As scintillator-based detectors are often inhomogeneous, analogue detection results in some detector areas producing a weaker signal from the photo-multiplier tube (PMT) for otherwise equal incident electron events [15–21]. An example PMT signal stream from an ADF detector may be seen in Fig. 1, where the electron event marked with A is too weak to be digitized with the threshold applied, whereas the slightly stronger signal for event B and much stronger signal for event C ensure that these events are correctly digitized. These irregularities in the detector surface can then make the quantification of analogue STEM images less reliable [1, 18, 19]. Although significant effort has been put in to remedy the inhomogeneities by statistically averaging them out and thereby determine parameters like sample thickness [14, 18, 22], digitization offer inherently quantitative images [9–10].

Coincidence loss occurs when two events occur too close together in time to be separated into two separate events [23–25]. The severity of the information loss depends on the arrival rate of the electrons at the detector and on the detector’s response to a single electron event. An electron event may be characterized by its rise time and decay time, which are the time it takes it to rise from 10% to 90% of its maximum intensity and decay from 90% to 10% respectively. Because a shorter rise time gives a higher gradient, a detector with a faster scintillator rise time will make it easier to separate two fast arriving electrons, while a longer rise time will make this more challenging [7]. Furthermore, a detector with a long decay time may obscure subsequent events in the decaying signal and therefore interfere with the appropriate detection of fast-arriving events [7]. By repeated simulation of the digitization of two sequential electron events with varying separations, it is possible to determine at what event separations correct digitization of the two events is statistically most likely for a specific electron pulse shape.

To realize faster counting rates with expanded dynamic range, the digitization process described by Peters *et al.*, relies on thresholding the gradient of the detector signal, as opposed to applying the threshold to the detector signal directly [10]. This means that the digitization hardware calculates the gradient of the detector signal, practically in real time, and applies a threshold in gradient space. A 16 µs excerpt from a pulse stream, and its corresponding gradient stream, from an ADF detector may be seen in Fig. 1. Events are counted where the gradient signal stays above the threshold for more than a given time-overthreshold, see for example the events marked with B, C, and D in Fig. 1. If the threshold is set too low, noise unavoidably present in the detector [26–28] will falsely be digitized as events. Conversely, if the threshold is set too high, the digitized signal will under-report the true signal as some weaker electron events may be missed. Setting the threshold at the optimal level, to maximize the number of correctly digitized electron events, can be very challenging due to the inhomogeneities and noise characteristics of the detector, yet this threshold profoundly defines the integrity of the digitized images acquired.

Here, we provide an automated and reproducible way of determining the optimal digitization threshold to apply. We show how this framework performs for both a single-channel monolithic detector and multi-channel signals from a segmented detector. Through an experimental threshold series for ADF imaging, we also show that our threshold optimization algorithm gives the threshold that will also optimize the CNR in the digitized ADF images. Although not demonstrated in this work, it is expected that this threshold optimization can also optimize the CNR of digitized images from a segmented detector.

## Threshold optimization for single-channel detector

Finding the threshold that yields the optimal CNR in the digitized image, manually is a challenging task. Since scintillator-based detectors are typically inhomogeneous, the amplitude of the signal from individual electron events can vary significantly, see Fig. 1. Additionally, most detectors have intrinsic noise originating from the stochastic processes of electron emission and detection as well as imperfections in the read-out system and amplification of the detected signal [26–28], which can occasionally make it difficult to distinguish weak electron events from noise. The digitization process used here implements a minimum time-over-threshold limit, demanding that the signal has to stay above the threshold for a configurable amount of time (e.g. 24 ns), to reduce the risk of counting random noise as events. Still, manual setting of the threshold on a noisy signal is inherently sensitive to human bias. Therefore, developing a method to automate the thresholding makes pulse counted ADF STEM a more robust and less subjective experimental technique.

**Fig. 2.**
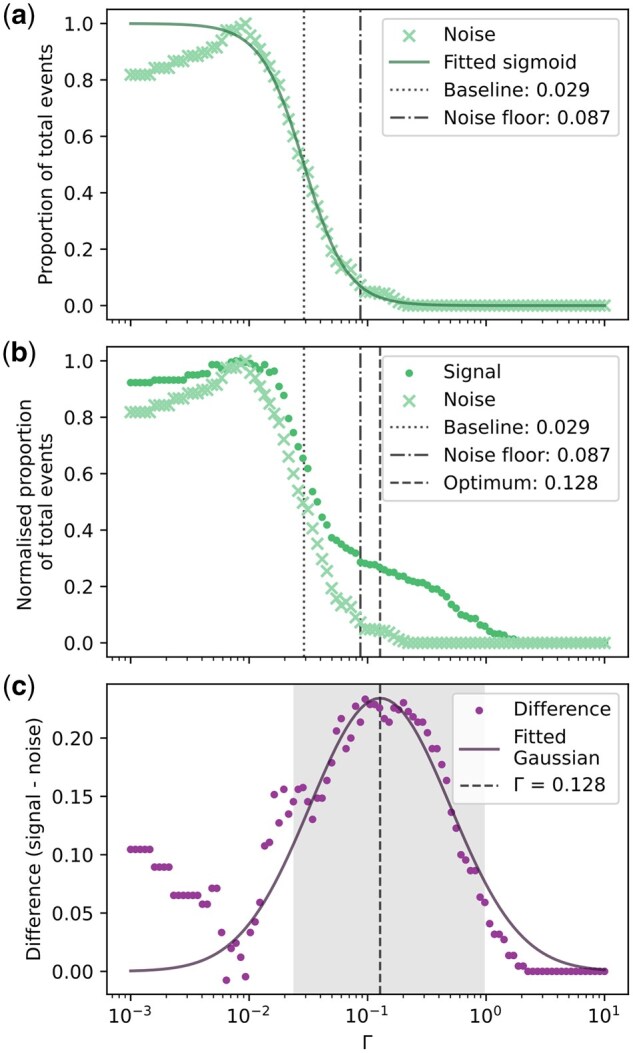
Optimization of digitization threshold for a monolithic ADF detector. (a) The number of events counted in a noise stream versus ln(Γ) with the best fit Sigmoid function to determine the baseline lower limit for the threshold. (b) Number of events counted in a noise stream (crosses) and a signal stream (dots) versus ln(Γ). The noise limit where number of events counted from the noise stream is less than 10 in the 80 µs streams, which equates to 1.25 × 10^5^ per second, is indicated as well as the optimal threshold. (c) The difference between normalized signal counts and noise counts. From this plot the optimal threshold is found by fitting a Gaussian to the shaded area, which contains the points in the neighbourhood of the maximum difference between signal counts and noise counts.

Not only does automation of the threshold setting make the electron counting technique more easily adoptable experimentally, but also yields more reliable and reproducible results. For optimizing the threshold the algorithm will have to know the noise signal from the detector in order to find the noise floor and baseline lower limit of the threshold. To calibrate this, a noise stream should be recorded with the detector inserted and the beam blanked, after the desired brightness and contrast level for the detector is set. Then a stream with electron events on the detector should be recorded with the same brightness and contrast levels. When setting the brightness and contrast for a detector before using a signal digitizer, the levels should be set such that no electron pulses are clipped in the pulse stream, in addition to making sure to not clip high or low intensity image values by investigating the image histograms [29]. The optimal threshold may be found by comparing the number of counts resulting from the noise stream and the signal stream at a number of different test thresholds. In this work, the experimental streams all have a duration of just over 80 µs, which illustrates how fast these streams are to acquire to optimize the threshold before the experiment. Using 100 different test thresholds, Γ, evenly spaced logarithmically in the range 10^−3^ to 10^1^ the noise stream and the signal stream are digitized. Figure 2a shows the proportion of the noise stream that have been counted as electron events as a function of the threshold applied plotted on a log-scale. Setting a very small threshold (Γ → 0), results in almost all the noise being falsely counted as electron events. While a higher time-over-threshold limit could filter out the noise, it may also filter out events if set too high.

Ideally, there would be no accidental electrons and the threshold could simply be set right above where noise starts being falsely counted as events. In reality, it is impossible to know exactly how many accidental electrons appear in a given noise stream, and we need to define a baseline lower limit for the threshold and an acceptable noise floor. Curve-fitting a semi-­empirical Sigmoid function to the noise count curve allows us to extract the inflection point as the baseline lower limit for the threshold for the detector in its current configuration. The threshold set for digitization of electron events from this detector should never go below this baseline lower limit. Further, we define a number of allowable event counts per time such that we can define a reasonable noise floor. Because accidental electrons may hit the detector [30], even when the beam is blanked, this is deemed a good compromise for defining the noise floor of the detector since allowing no accidental electron events would lead to mistakenly setting the noise floor too high. An allowable event count for the noise stream is set to 10 events for the full 80 µs stream, which is roughly 1 false event per 1000 sampling points. This noise floor works out to be where less than 0.125 electrons hit the detector per microsecond, which is equivalent to just over one electron per 10 µs. For comparison, a full 512 by 512 pixel image typically contains roughly 60 000 electrons.

Similar to how setting the threshold too low yields many false positives, setting the threshold too high results in no electron counts at all. We seek to identify the optimal threshold somewhere in between. Comparing the number of counts versus Γ for the noise stream and signal streams, see Fig. 2b, reveals that there is a quite significant overlap between the high number of counts for the signal stream and the occurrence of a significant amount of counts for the noise stream. The optimal threshold will be the threshold which minimizes the number of actual electron events that are missed (false negatives), and simultaneously minimizes the number of noise accidentally counted as events (false positives). Taking the difference between the normalized number of events counted for the noise stream and the signal stream gives the difference graph in Fig. 2c. Maximizing this difference should give the highest possible number of real positives, whilst minimizing the number of false positives. In order to robustly determine the maximum of the difference, we fit a Gaussian function to the shaded area of the difference plot, see Fig. 2c. The threshold corresponding to the maximum of this Gaussian will be taken as the optimal threshold because it represents the optimal middle-ground where both the number of false positives and number of false negatives are minimized. Note that the noise stream and the signal stream being used for this optimization process are recorded prior to the threshold optimization process such that the threshold applied to the streams is the only thing changing for the graphs in Fig. 2.

## Importance of threshold optimizing for ADF experimentally

**Fig. 3.**
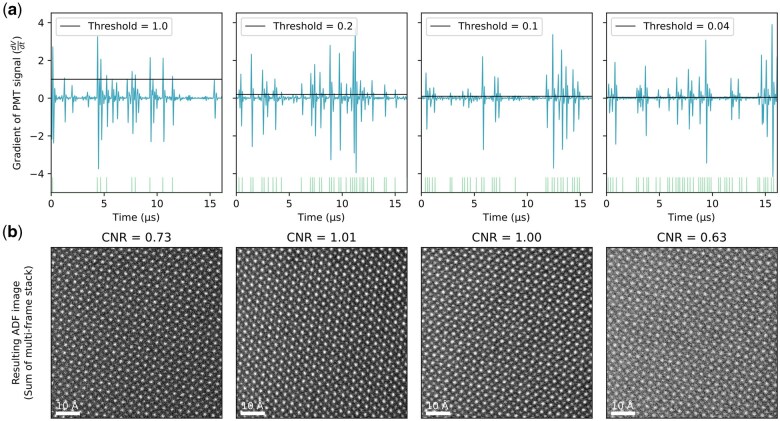
Experimental results with varying thresholds. (a) Gradient of PMT signal from the ADF detector and the applied threshold. For visibility only part of the total stream captured is shown. (b) Resulting digital ADF images of STO from summing a multi-frame stack with 100 frames when the above thresholds have been applied to the gradient of the analogue ADF signal for the experimental digitization. The dwell time used was *δ_t_* = 100 ns, beam current *I * =  8 pA, and semi-convergence angle *α * =  30 mrad. The CNR value was calculated by comparing the Sr and O columns.

Using the optimal threshold is vital for achieving reliable and high quality digitized ADF images since it maximizes the number of correctly counted electrons. The images of SrTiO_3_ (STO) from using four different thresholds to digitize the signal from an ADF detector can be seen in Fig. 3. An expanded series of these ADF images and their Gaussian filtered versions may be seen in the Supplementary data. These images were acquired as a multi-frame stack of 100 frames, with pixel dwell time of *δ_t_* = 100 ns, beam current *I * =  8 pA, and semi-convergence angle *α *= 30 mrad. The CNR in these images is obtained as


(1) CNR=|μ1-μ2|σ12+σ22,

where *µ*_1_ and *σ*_1_ are the mean and standard deviation of Sr columns and *µ*_2_ and *σ*_2_ are the mean and standard deviation of O columns in the ADF images [31–32]. Note that CNR is used here rather than signal-to-noise ratio (SNR) because of its more straightforward interpretation and higher robustness.

**Fig. 4.**
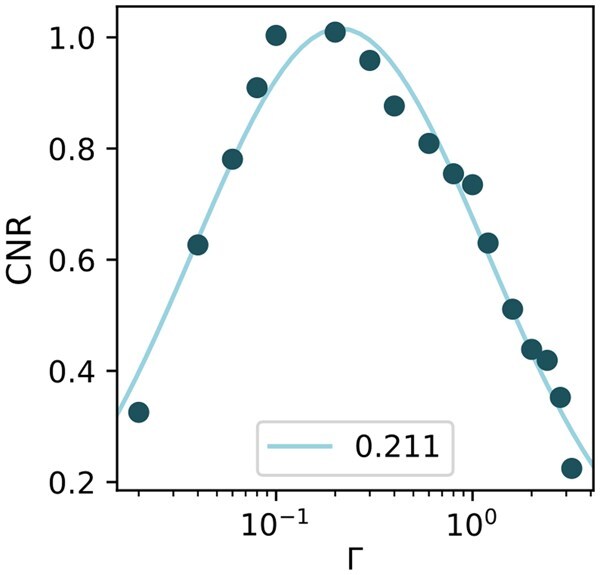
Contrast-to-noise ratio between Sr and O columns as a function of threshold for experimental ADF images.

From the CNR values for these images we see that the optimal threshold likely lie between 0.1 and 0.2 for this specific experiment, since these two thresholds give the highest CNR values, suggesting that the optimal threshold is found in the range between them. Fitting a Gaussian to the CNR values for all the experimentally tested thresholds gives a peak around Γ = 0.211, see Fig. 4, indicating that the optimal threshold might lie slightly higher, but this is likely to be experiment-specific. A similar analysis using the structural similarity between the ADF images Gaussian filtered with *σ* = 2 px and the raw ADF images may be seen in Fig. S1. In general, as the threshold is lowered, more false positives are seen because noise is being falsely detected as electron events. This increase in the number of false positives results in a lower CNR as can be seen for the ADF image digitized with Γ = 0.04 in Fig. 3b. Conversely, for higher thresholds, many electron events are missed giving a lower CNR due to an elevated number of false negatives.

While the optimal threshold could be found by maximizing the CNR in the final images, this is very time-consuming and involves unnecessarily exposing the sample to the beam. By using only two streams (one noise stream and one signal stream) of 80 µs, the optimal threshold can be found efficiently. Comparing the estimated optimal threshold for this data set, which was found to be Γ = 0.128, with the experimental images suggesting the maximum CNR is found with the threshold around 0.2 indicates that our method adequately estimates the threshold that will yield the maximum CNR in the final digitized images.

## Optimizing threshold for segmented detectors

When using segmented detectors, typically for phase contrast or retrieval techniques, it is vital that the individual segments are standardized to give the same intensity range output for the same signal level to avoid any false skewness in the data acquired [33]. Standardization means that when the detector is uniformly illuminated the intensity level should be the same in all segments. This is achieved by setting the contrast and brightness values for each segment independently, and iteratively checking the intensity level achieved for all the segments to fulfil the equality requirement for the analogue signal. Then the optimal threshold for digitization may be found in the same way as for a monolithic detector by acquiring noise streams with the beam blanked and signal streams with the beam on. The streams will need to be acquired for all the segments that are to be used for the experiment and should all be acquired simultaneously to ensure all segments as treated equally.

**Fig. 5.**
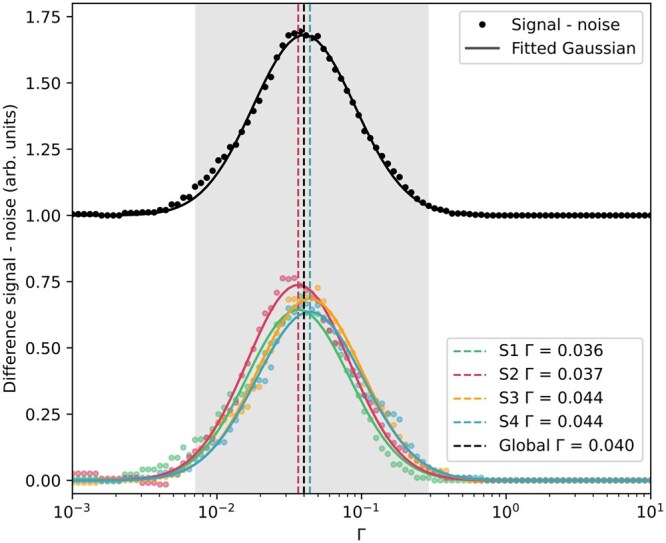
Difference between the signal counts and noise counts for four segments separately (colours) and all summed together (black). Fitting a Gaussian to the global difference (black), gives an optimized threshold of Γ = 0.040. This value is close to the optimal threshold for S3 and S4, but a bit higher than the optimal threshold for S1 and S2, as may be seen from the horisontal dotted lines.

By summing all the noise counts and the signal counts for the four segments and then fitting a Gaussian to the difference between these the global optimal threshold (peak of the Gaussian in the neighbourhood of the maximum difference) may be found at Γ = 0.040, see Fig. 5. From a comparison of this global optimal Γ with the optimal Γ from the difference graphs for the individual segments it is evident that the global optimum is close to optimal for S3 and S4, and slightly too high for S1 and S2. Thus, it is recommended that the optimal thresholds are found and set individually per segment, to be based on their individual noise characteristics and electron event responses, since both noise and signal streams have to be acquired for all segments regardless.

**Fig. 6.**
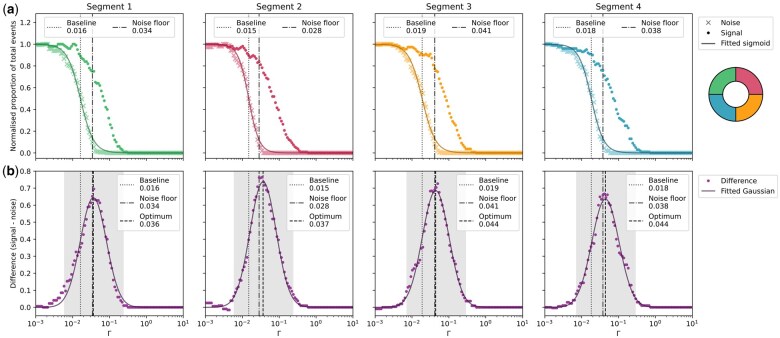
Threshold optimization for four segments on a SAAF detector. (a) Counts in the noise stream (crosses) and signal stream (dots) when digitized with various thresholds. The Sigmoid function fitted to the noise data yields the baseline for the lowest possible threshold at its inflection point, and the noise floor is defined as where just over one electron hits the detector segment per 10 µs. (b) Difference in normalized counts between the signal and noise streams, with a fitted Gaussian yielding the optimal threshold at its peak. Note that the optimal threshold is estimated to be very close to the noise floor for segment S4, illustrating the need for an unbiased and automated way of determining these thresholds.

The data used to optimize the threshold for four segments on a segmented annular all-field (SAAF) detector [33] can be seen in Fig. 6. These results illustrate how the threshold should be optimized on a per-segment basis, since two of the segments have lower optimal thresholds when compared with the two other segments. Note also that the range of thresholds yielding multiple false positives whilst still having a significant amount of false negatives is more pronounced for this detector when compared with the monolithic detector. While there may be several different reasons for this, the most important seems to be the high noise-level present in the streams from the segments of this detector, see example signal and noise streams in Fig. S2. Being a 16-segmented detector, it is possible that the occurrence of electron events in any of the other 15 segments can cross over and disturb the PMT signal for any one segment even though we only used and digitized the signal from four segments for this work. There are many potential sources that could lead to cross-talking, including electrons hitting the edge between two segments, re-emission of secondary electrons from the detector itself, light leakage from one segment to another in the light-guides from the scintillator to the PMT, and electrical disturbance between the individual segment cables. The presence of cross-talking between segments can make it difficult to distinguish true electron events from the segment itself, from cross-talking events and other noise present. To show the effect the thresholding of the signal from the SAAF detector can have on the images, the resulting segment and COM images from using two different thresholds for the digitization can be seen in Fig. S3.

Importantly, losing signal to coincidence loss is a factor that should be accounted for with digitization, since the arrival of two electrons too close together in time to be separated into two events in the digitization process does cause loss of information. Additionally, coincidence loss is much more important to keep in mind when placing the detector in the bright field region, since the intensity collected with a detector decreases exponentially with increasing inner collection angle meaning that the current delivered to the detector in the ABF region is much higher than for ADF [34]. Therefore, the beam current must be lowered when digitizing the signal from a detector in the ABF region in order to reduce the arrival rate of electrons and avoid coincidence loss. Naturally, the segmentation of the detector helps to reduce coincidence loss by dividing the current between the segments, however with higher beam current the probability of multiple electrons arriving too close to be successfully separated is still increased. By simulation we have found that an electron is less than 5% likely to be lost to coincidence loss if it arrives more than 0.174 ± 0.004 µs after the previous electron for the ADF detector and 0.37 ± 0.03 µs for the SAAF detector. This translates to a current of 0.92 ± 0.02 pA delivered to the detector for the ADF detector, and 0.43 ± 0.04 pA delivered to each segment for the SAAF detector. Note that the current delivered to the detector is related to the beam current through the scattering characteristics of the sample and the detection area of the detector, determined by its geometry and the inner and outer collection angles, and that coincidence loss may never be fully eliminated due to the Poissonian nature of electron emission and, consequently, detection. Figure 1 shows an example pulse stream from the ADF detector, including an example of coincidence loss (marked by D). Since detectors with a shorter scintillation rise-time produce signals that are easier to digitize [7], due to the sharper onset giving a higher gradient, the development of scintillator-based detectors that have a fast response and low cross-talking can allow higher beam currents for segmented detectors in the ABF region. Combined with our unbiased framework for optimization of the threshold, digitization of the signal from these detectors can help advance ultra low-dose and fast real-space frame-rate phase contrast imaging.

## Conclusion

We have introduced a less biased and more reproducible way of choosing the optimal threshold for digitization of the analogue signal from monolithic and segmented detectors in STEM by development of a framework for automatic optimization of digitization threshold. The optimization algorithm relies on two streams of data, namely a stream of the noise in the detector, with the beam blanked, and a stream with actual signal. Experimentally, these streams are easy and fast to acquire. We show that the more noise is present in the streams from the detector, the more difficult it is to set the threshold adequately—exacerbating the need for a threshold optimizer to minimize the number of false negatives and false positives at the same time.

The framework presented in this work tests a number of different thresholds on the two streams and finds the threshold that maximizes the difference between the number of counts in the signal stream and noise stream. Our experimental images suggest that the optimal threshold found from this procedure coincides well with the threshold that gives the highest CNR in the digitized image for a monolithic ADF detector. It is expected, although not demonstrated here, that the proposed method for threshold optimization may also optimize the CNR for images acquired and reconstructed from a segmented detector signal. With this automatic threshold optimizer, digitization of detector signals becomes more reliable and yields more reproducible results by reducing the susceptibility to human bias.

## Supplementary Material

dfaf025_Supplementary_Data
