# Supplementary material for: Auto-thresholding for unbiased electron counting
Source: Microscopy (Oxf). 2025 May 19;74(6):404–10. doi: 10.1093/jmicro/dfaf025 (PMC12685148; doi:10.1093/jmicro/dfaf025)
Supplement: dfaf025_Supplementary_Data [file dfaf025_supplementary_data.pdf]

# Supplementary Information: Auto-thresholding for Unbiased Electron Counting

Julie Marie Bekkevold[1,2,*], Jonathan J. P. Peters[1,2], Ryo Ishikawa[3], Naoya Shibata[3], and Lewys Jones[1,2]

[1] *School of Physics, Trinity College Dublin, College Green, Dublin D02 PN40, Ireland*
[2] *Advanced Microscopy Laboratory, Centre for Research on Adaptive Nanostructures and Nanodevices (CRANN), Trinity College Dublin, Dublin D02 DA31, Ireland*
[3] *Institute of Engineering Innovation, University of Tokyo, Bunkyo, Tokyo 113-8656, Japan*

1st May 2025

## S1 Additional Details on the Digitisation Process

The digitisation of the signal from the detector used in this work relies on counting electron events through thresholding the gradient of the intensity signal output from the detector. This method has been extensively reported by T. Mullarkey and J.J.P. Peters in [1–3]. Analog scintillator-based detectors output a voltage signal from the photo-multiplier tube (PMT). The digitisation hardware samples this analog output at 125 Msps, calculates its gradient in real-time, and applies a threshold to the gradient signal. Every occurrence of the gradient staying above the threshold for more than the pre-defined time-over-threshold is counted as an electron.

## S2 Structural Similarity Index (SSIM) for Digitised ADF Images

In addition to the contrast-to-noise ration (CNR), the quality of the digitised ADF images with different thresholds have also assessed using the structural similarity index (SSIM). This

image quality metric provides a more holistic assessment of the image quality by taking the image luminance, contrast, and structure into account. As described by Z. Wang et. al. [4, 5], the SSIM is given by

$$\mathrm{SSIM}(x, y) = l(x, y) \cdot c(x, y) \cdot s(x, y). \tag{1}$$

The luminance, $l(x, y)$, contrast, $c(x, y)$, and structure, $S(x, y)$ are defined as

$$l(x, y) = \frac{2\mu_x \mu_y + C_1}{\mu_x^2 + \mu_y^2 + C_1} \tag{2}$$

$$c(x, y) = \frac{2\sigma_x \sigma_y + C_2}{\sigma_x^2 + \sigma_y^2 + C_2} \tag{3}$$

$$s(x, y) = \frac{\sigma_{xy} + C_3}{\sigma_x \sigma_y + C_3} \tag{4}$$

(5)

where $\mu_x$ and $\sigma_x$ denotes the mean intensity and variance of signal $x$, and $\sigma_{xy}$ is the co-variance between signal $x$ and $y$. The constants $C_1, C_2, C3 \geq 0$ are included to avoid instability when $\mu$- and $\sigma$-values are close to zero. Here, the SSIM is calculated between each image and a smoothened version of itself. The smoothening has been applied using a Gaussian filter with $\sigma = 2$ px.

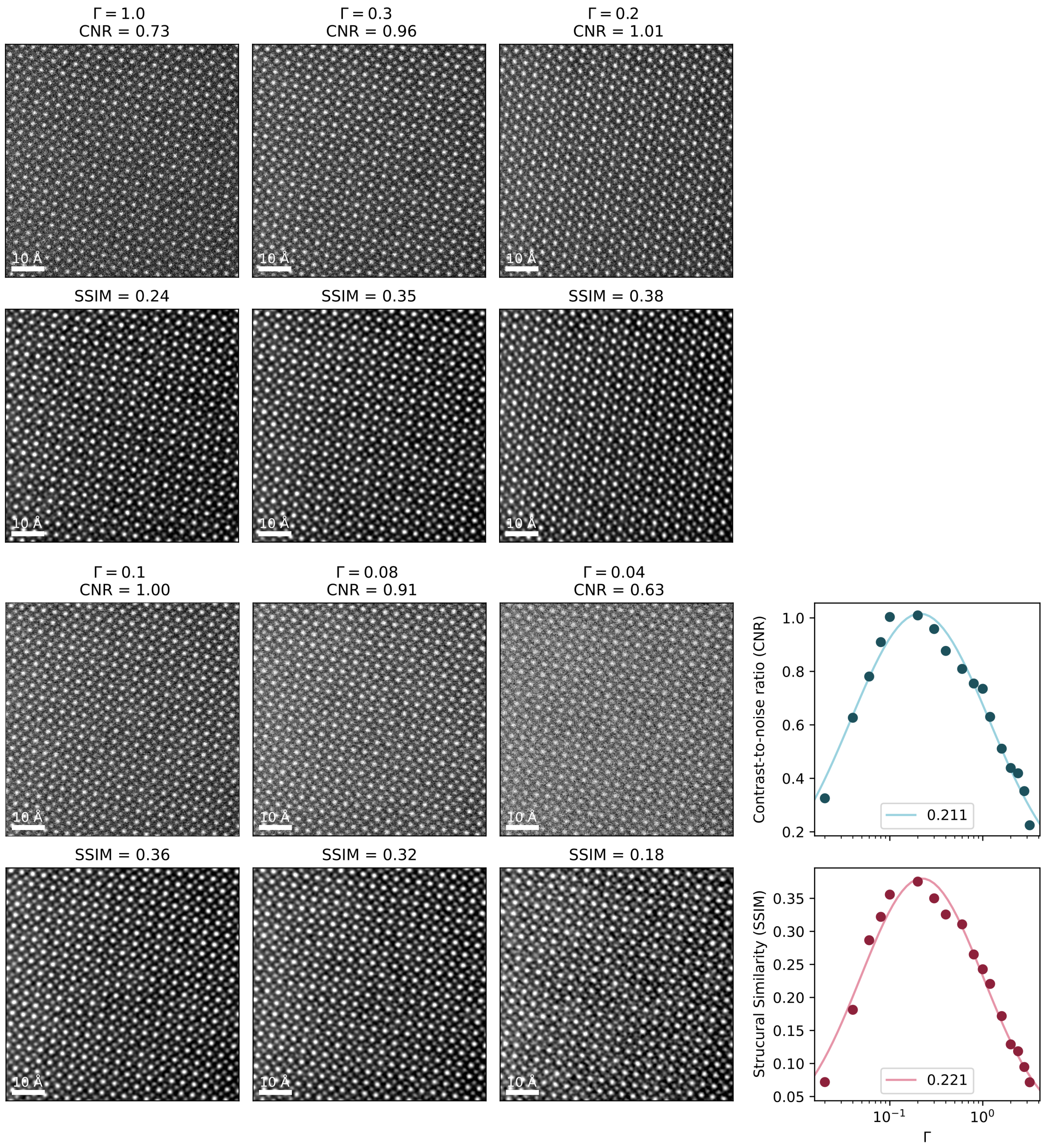


**Figure S1:** Expanded series of ADF images, sum of 100 frames, raw (top) and Gaussian filtered with $\sigma = 2$ px (bottom). The CNR is calculated by comparing Sr and O columns, and the SSIM is the structural similarity between the filtered and the raw image. The plots show the CNR and the SSIM for images digitised with all the different experimentally tested thresholds, with a Gaussian fit for which the legend indicates the peak.

## S3 Additional Segmented Detector Data

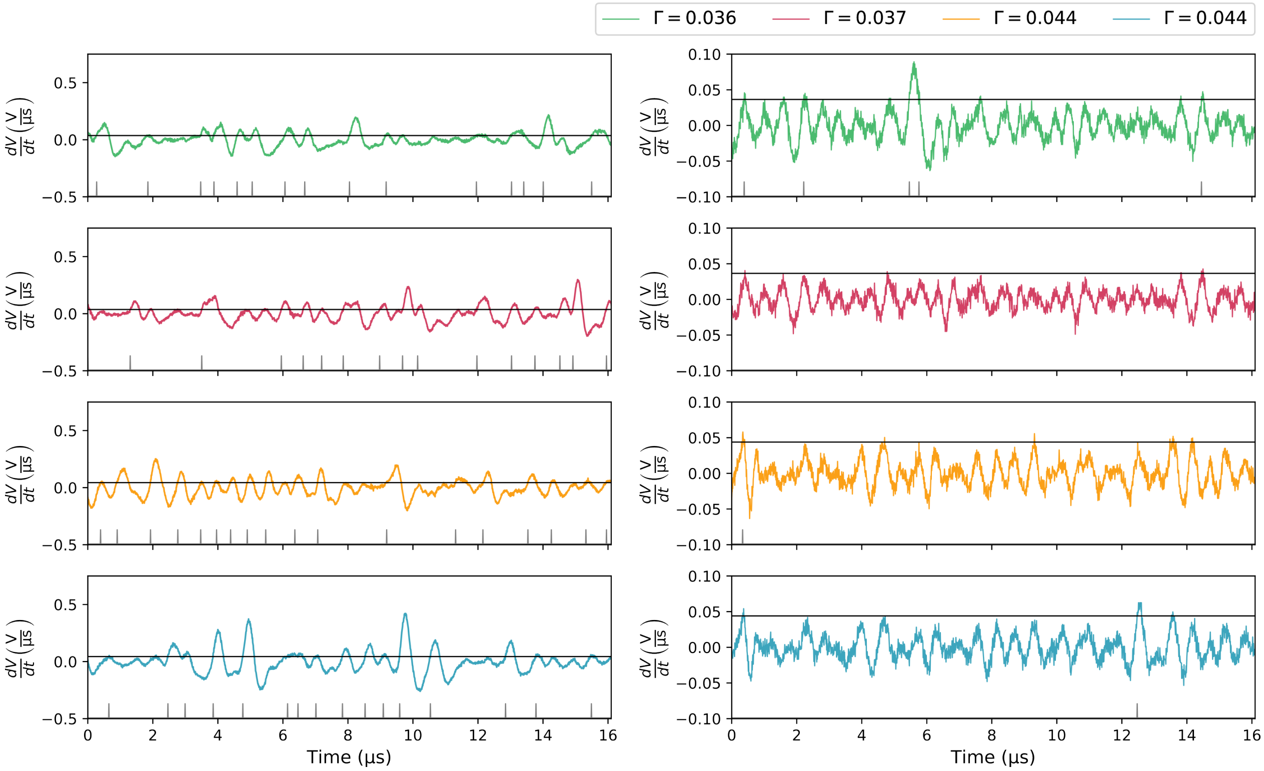


**Figure S2:** Gradient of signal streams (left) and noise streams (right) for four segments on a SAAF detector. The digitised signal when using the optimised threshold, indicated in the legend, for each channel is indicated at the bottom.

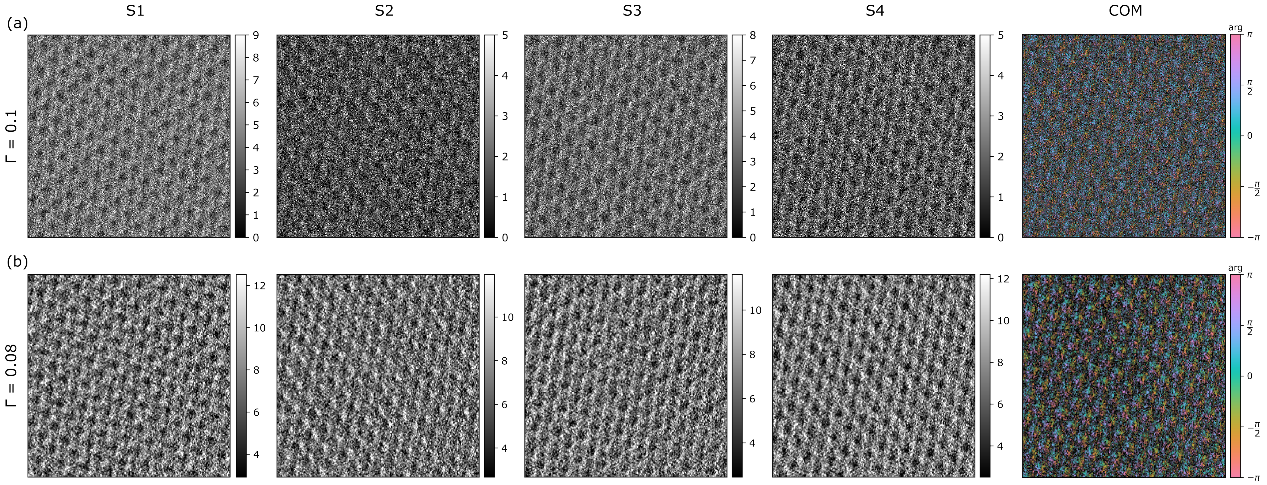


**Figure S3:** Segment images and the resulting COM from segmented detector signal digitised with (a) $\Gamma = 0.1$ and (b) $\Gamma = 0.08$. Note how the lower threshold yields a significant increase in the number of electrons counted. The images have been cropped to 256 by 256 pixels from their full acquired size at 512 by 512 pixels. Acquisition parameters were $I = 5.2$ pA, $\alpha = 30$ mrad, and $\delta_t = 0.1$ µs.

## References


[1] Tiarnan Mullarkey, Clive Downing, and Lewys Jones. "Development of a Practicable Digital Pulse Read-Out for Dark-Field STEM". In: *Microsc Microanal* 27.1 (2021), pp. 99–108. DOI: 10.1017/S1431927620024721.

[2] Tiarnan Mullarkey et al. "How Fast is Your Detector? The Effect of Temporal Response on Image Quality". In: *Microscopy and Microanalysis* 29.4 (2023), pp. 1402–1408. DOI: 10.1093/micmic/ozad061.

[3] Jonathan J. P. Peters et al. "Electron counting detectors in scanning transmission electron microscopy via hardware signal processing". In: *Nat Commun* 14.1 (2023). Number: 1 Publisher: Nature Publishing Group, p. 5184. DOI: 10.1038/s41467-023-40875-w.

[4] Zhou Wang, A.C. Bovik, H.R. Sheikh, and E.P. Simoncelli. "Image quality assessment: from error visibility to structural similarity". In: *IEEE Transactions on Image Processing* 13.4 (2004), pp. 600–612. DOI: 10.1109/TIP.2003.819861.

[5] Zhou Wang and Alan C. Bovik. "Mean squared error: Love it or leave it? A new look at Signal Fidelity Measures". In: *IEEE Signal Processing Magazine* 26.1 (2009), pp. 98–117. DOI: 10.1109/MSP.2008.930649.


## List of Figures


S1 Expanded series of ADF images, sum of 100 frames, raw (top) and Gaussian filtered with $\sigma = 2$ px (bottom). The CNR is calculated by comparing Sr and O columns, and the SSIM is the structural similarity between the filtered and the raw image. The plots show the CNR and the SSIM for images digitised with all the different experimentally tested thresholds, with a Gaussian fit for which the legend indicates the peak. . . . . . . . . . . . . . . . . . . . . . . . 3

S2 Gradient of signal streams (left) and noise streams (right) for four segments on a SAAF detector. The digitised signal when using the optimised threshold, indicated in the legend, for each channel is indicated at the bottom. . . . . 4
S3 Segment images and the resulting COM from segmented detector signal digitised with (a) $\Gamma = 0.1$ and (b) $\Gamma = 0.08$. Note how the lower threshold yields a significant increase in the number of electrons counted. The images have been cropped to 256 by 256 pixels from their full acquired size at 512 by 512 pixels. Acquisition parameters were $I = 5.2$ pA, $\alpha = 30$ mrad, and $\delta_t = 0.1$ µs. . . 5
